# Phylogenomics of *Xanthomonas* field strains infecting pepper and tomato reveals diversity in effector repertoires and identifies determinants of host specificity

**DOI:** 10.3389/fmicb.2015.00535

**Published:** 2015-06-03

**Authors:** Allison R. Schwartz, Neha Potnis, Sujan Timilsina, Mark Wilson, José Patané, Joaquim Martins, Gerald V. Minsavage, Douglas Dahlbeck, Alina Akhunova, Nalvo Almeida, Gary E. Vallad, Jeri D. Barak, Frank F. White, Sally A. Miller, David Ritchie, Erica Goss, Rebecca S. Bart, João C. Setubal, Jeffrey B. Jones, Brian J. Staskawicz

**Affiliations:** ^1^Department of Plant and Microbial Biology, University of California, BerkeleyBerkeley, CA, USA; ^2^Department of Plant Pathology, University of FloridaGainesville, FL, USA; ^3^Donald Danforth Plant Science CenterSt. Louis, MO, USA; ^4^Department of Biochemistry, Institute of Chemistry, University of São PauloSão Paulo, Brazil; ^5^Department of Plant Pathology, Kansas State UniversityManhattan, KS, USA; ^6^School of Computing, Federal University of Mato Grosso do SulCampo Grande, Brazil; ^7^Gulf Coast Research and Education Center, University of FloridaWimauma, FL, USA; ^8^Department of Plant Pathology, University of Wisconsin, MadisonMadison, WI, USA; ^9^Department of Plant Pathology, Ohio Agricultural Research and Development CenterWooster, MA, USA; ^10^Department of Plant Pathology, NC State UniversityRaleigh, NC, USA; ^11^Virginia Bioinformatics Institute, Virginia TechBlacksburg, VA, USA

**Keywords:** *Xanthomonas*, type III effector repertoire, phylogenomics, host specificity, bacterial spot disease, AvrBsT, XopQ

## Abstract

Bacterial spot disease of pepper and tomato is caused by four distinct *Xanthomonas* species and is a severely limiting factor on fruit yield in these crops. The genetic diversity and the type III effector repertoires of a large sampling of field strains for this disease have yet to be explored on a genomic scale, limiting our understanding of pathogen evolution in an agricultural setting. Genomes of 67 *Xanthomonas euvesicatoria* (*Xe*), *Xanthomonas perforans* (*Xp*), and *Xanthomonas gardneri* (*Xg*) strains isolated from diseased pepper and tomato fields in the southeastern and midwestern United States were sequenced in order to determine the genetic diversity in field strains. Type III effector repertoires were computationally predicted for each strain, and multiple methods of constructing phylogenies were employed to understand better the genetic relationship of strains in the collection. A division in the *Xp* population was detected based on core genome phylogeny, supporting a model whereby the host-range expansion of *Xp* field strains on pepper is due, in part, to a loss of the effector AvrBsT. *Xp*-host compatibility was further studied with the observation that a double deletion of AvrBsT and XopQ allows a host range expansion for *Nicotiana benthamiana*. Extensive sampling of field strains and an improved understanding of effector content will aid in efforts to design disease resistance strategies targeted against highly conserved core effectors.

## Introduction

Species of *Xanthomonas* cause bacterial spot disease on cultivated pepper (*Capsicum annuum*) and tomato *(Solanum lycopersicum)* and are the most devastating to crops grown in warm, humid climates such as in the southeastern and midwestern United States (Obradovic et al., 2008). Once considered a single species, *Xanthomonas vesicatoria* infecting pepper and tomato has been reclassified several times (Stall et al., 1994; Vauterin et al., 1995; Jones and Stall, 2000), but was most recently separated into four distinct species: *X. euvesicatoria* (*Xe*), *X. vesicatoria* (*Xv*), *X. perforans* (*Xp*), and *X. gardneri* (*Xg*) (Jones et al., 2004). While *X*e, *Xg*, and *Xv* infect both pepper and tomato, *Xp* has only been reported on tomato. Although the four pathogens are present and destructive on a global scale (Jones et al., 1998a; Timilsina et al., 2015), the history and distribution of *Xe*, *Xp*, and *Xg* has changed dramatically in the United States, particularly with the emergence of *Xp* as the dominant tomato pathogen over *Xe* in Florida beginning in the early 1990's (Jones et al., 1998b; Tudor-Nelson et al., 2003; Hert et al., 2005; Stall et al., 2009; Horvath et al., 2012) and *Xg* as a major tomato pathogen in Ohio and Michigan beginning in 2009 (Ma et al., 2011). Outbreaks of *Xv* have not been reported in the United States (Timilsina et al., 2015).

Different phylogenetic analyses found a close evolutionary relationship between *Xe* and *Xp* in comparison to *Xg* and *Xv* (Young et al., 2008; Parkinson et al., 2009; Almeida et al., 2010; Hamza et al., 2010; Midha and Patil, 2014). Comparative genomics of reference strains Xe85-10 (Thieme et al., 2005) with Xp91-118, Xg101, and Xv1111 (Potnis et al., 2011) provided the first insights into the shared and unique virulence factors of these pepper and tomato pathogens. A major factor contributing to the virulence and host specificity of these pathogens is the repertoire of effectors secreted into the host plant cell via the type III secretion system (Grant et al., 2006). Xanthomonads have evolved effectors with diverse mechanisms to promote virulence, even adopting processes specific to eukaryotes (Kay and Bonas, 2009). The recognition of specific effector proteins by specific cognate resistance (R) proteins leads to defense responses that have been termed Effector Triggered Immunity (ETI), which is accompanied by localized cell death, associated tissue collapse known as the hypersensitive response (HR) at the site of infection, and limited spread of the pathogen (Jones and Dangl, 2006). Several type III effectors are conserved across multiple species and referred to here as core effectors. An additional variable set of effectors may provide specialization to specific hosts and cultivars (Hajri et al., 2009).

The deployment of R proteins in crops that can recognize and respond to core effectors is a potentially durable disease resistance strategy, depending on the evolutionary stability of the targeted cognate effector (Boyd et al., 2013). Because xanthomonads display relatively high genome plasticity, a more comprehensive understanding of the genetic diversity of pepper and tomato pathogens, with specific emphasis on effectors, is necessary for designing informed disease resistance strategies for agricultural areas afflicted by bacterial spot disease (Thieme et al., 2005; Potnis et al., 2011; Timilsina et al., 2015). A comparative genomic analysis considering many strains from a given geographic region over time will provide a representative view of the effectors present in the regional bacterial population and add insight into the evolutionary trends of effectors, and thus their potential usefulness as targets for R-gene mediated resistance strategies.

To this end we sequenced the genomes of 32 *Xp*, 25 *Xe*, and 10 *Xg* field strains that were collected from diseased peppers and tomatoes in the southeastern and midwestern United States. Here we describe the genetic diversity within and between species using core protein-coding genome phylogeny and whole genome single nucleotide polymorphism (SNP) analysis and present the computationally predicted type III effector repertoires of strains in our collection. The role played by the effectors AvrBsT and XopQ as host specificity determinants for *Xp* infecting pepper and *Nicotiana benthamiana* was also characterized.

## Materials and methods

### *Xanthomonas* strain collection

*Xe*, *Xp*, and *Xg* strains were collected from diseased tomatoes and peppers in the United States (Table 1). *Xp* strains were collected between 1998 and 2013 in Florida and Georgia. *Xg* strains were collected in Ohio and Michigan between 2010 and 2012. *Xe* strains were collected between 1994 and 2012 in Florida, North Carolina, Georgia, and Kentucky.

**Table 1:** **Summary of *Xanthomonas* field strains sequenced in this paper**.

**Species**	**Strain name**	**Origin in US**	**Host isolated**	**Year isolated**	**Isolation ID**	**Collector**
*X. euvesicatoria*	Xe073	North Carolina	P	1994	181	DR
	Xe074	Raleigh, NC	P	1994	199	DR
	Xe075	Soutwest FL	P	1995	206	DR
	Xe076	Naples, FL	P	1995	259	DR
	Xe077	Kentucky	P	1996	315	DR
	Xe078	Clewiston, FL	P	1997	329	DR
	Xe079	Jupiter, FL	P	1998	354	DR
	Xe081	Ft. Pierce, FL	P	1995	376	DR
	Xe082	Southeast FL	P	1998	455	DR
	Xe083	Belle Glade, FL	P	1999	490	DR
	Xe085	Boynton Beach, FL	P	1999	515	DR
	Xe086	Delray Beach, FL	P	2000	526	DR
	Xe091	Boca Raton, FL	P	2003	586	DR
	Xe101	Sampson Co., NC	P	2008	678	DR
	Xe102	Manetee, FL	P	2008	679	DR
	Xe103	Pender Co., NC	P	2009	681	DR
	Xe104	Sampson Co., NC	P	2010	683	DR
	Xe105	Granville, NC	P	2010	684	DR
	Xe106	Granville, NC	P	2010	685	DR
	Xe107	Granville, NC	P	2011	689	DR
	Xe108	Pender Co., NC	P	2012	695	DR
	Xe109	Cook Co., GA	P	2004	F4-2	DR
	Xe110	Tift Co. GA	P	2004	G4-1	DR
	Xe111	Colquitte Co., GA	P	2004	H3-2	DR
	Xe112	Brooks Co., GA	P	2004	L3-2	DR
*X. perforans*	Xp4B	Citra, FL	T	1998	Xp4B	JJ
	Xp2010	Hendry County, FL	P	2010	Xp2010	JJ
	TB6	Hillsborough, FL	T	2013	TB6	JJ
	TB9	Hillsborough, FL	T	2013	TB9	JJ
	TB15	Hillsborough, FL	T	2013	TB15	JJ
	Xp3-15	Decatur Co., GA	T	2006	Xp3-15	JJ
	Xp4-20	Decatur Co., GA	T	2006	Xp4-20	JJ
	Xp5-6	Decatur Co., GA	T	2006	Xp5-6	JJ
	Xp7-12	Manatee Co., FL	T	2006	Xp7-12	JJ
	Xp8-16	Manatee Co., FL	T	2006	Xp8-16	JJ
	Xp9-5	Manatee Co., FL	T	2006	Xp9-5	JJ
	Xp10-13	Manatee Co., FL	T	2006	Xp10-13	JJ
	Xp11-2	Palm Beach Co, FL	T	2006	Xp11-2	JJ
	Xp15-11	Miami-Dade Co., FL	T	2006	Xp15-11	JJ
	Xp17-12	Collier Co., FL	T	2006	Xp17-12	JJ
	Xp18-15	Collier Co., FL	T	2006	Xp18-15	JJ
	GEV839	Hardee Co., FL	T	2012	GEV839	JJ
	GEV872	Immokalee, FL	T	2012	GEV872	JJ
	GEV893	Collier Co.	T	2012	GEV893	JJ
	GEV904	Hillsborough, FL	T	2012	GEV904	JJ
	GEV909	Collier Co.	T	2012	GEV909	JJ
	GEV915	Hillsborough, FL	T	2012	GEV915	JJ
	GEV917	Hillsborough, FL	T	2012	GEV917	JJ
	GEV936	Lee, FL	T	2012	GEV936	JJ
	GEV940	GCREC, FL	T	2012	GEV940	JJ
	GEV968	Manatee Co., FL	T	2012	GEV968	JJ
	GEV993	Hendry Co., FL	T	2012	GEV993	JJ
	GEV1001	Quincy, FL	T	2012	GEV1001	JJ
	GEV1026	West Coast, FL	T	2012	GEV1026	JJ
	GEV1044	Collier Co., FL	T	2012	GEV1044	JJ
	GEV1054	Manatee Co., FL	T	2012	GEV1054	JJ
	GEV1063	Collier Co., FL	T	2012	GEV1063	JJ
*X. gardneri*	Xg153	Gibsonburg, OH	T	2010	SM194-10	SM
	Xg156	Blissfield, MI	T	2010	SM177-10	SM
	Xg157	Blissfield, MI	T	2010	SM182-10	SM
	Xg159	Blissfield, MI	T	2010	SM220-10	SM
	Xg160	Blissfield, MI	T	2010	SM234-10	SM
	Xg164	Ottawa, OH	T	2011	SM406-11	SM
	Xg165	Ottawa, OH	T	2011	SM413-11	SM
	Xg173	Unknown, OH	T	2011	SM605-11	SM
	Xg174	Wayne Co., OH	T	2012	SM775-12	SM
	Xg177	Sandusky Co., OH	T	2012	SM795-12	SM

*Year, host, location, and isolation source are described. DR, David Ritchie; JJ, Jeffrey Jones; SM, Sally Miller*.

### Genome sequencing and effector predictions

Bacterial genome sequencing and effector prediction were completed as previously described (Bart et al., 2012). Briefly, genomic DNA was isolated with a modified CTAB protocol and prepared for library construction and sequencing on the Illumina platforms. Ten *Xg* libraries were pooled into a single lane of MiSeq (PE250). *Xe* and the *Xp* strains from 2006 were sequenced by multiplexing 48 libraries per lane on an Illumina HiSeq 2000 sequencer (PE100). The *Xp* strains from 2012 were sequenced by multiplexing 20 libraries per lane on an Illumina MiSeq (PE150). Genomic *de novo* assemblies were constructed using CLC Genomics Workbench using a length fraction of 0.9 and a similarity of 1.0. Potential effectors were identified by an in-house Python script utilizing BLAST against a database of known effectors, using a filter of greater than 45% amino acid similarity over 80% of the length of the target sequence (Bart et al., 2012).

### Phylogenomic inference using core protein-coding genes

All genomes sequenced in this study were annotated using the National Center for Biotechnology Information Prokaryotic Genome Annotation Pipeline (PGAP) (http://www.ncbi.nlm.nih.gov/books/NBK174280). Ortholog families were determined using the GET_HOMOLOGUES package (Contreras-Moreira and Vinuesa, 2013), which includes a step of all-against-all BlastP (Altschul et al., 1997) followed by clustering based on OrthoMCL to yield homologous gene clusters (Li et al., 2003). This result was filtered using compare_cluster.pl (a script in the GET_HOMOLOGUES package) with option “-t n,” where *n* is the number of genomes, keeping only the gene families that have exactly one representative from each genome considered; the protein-coding genes in these families were considered the “core genome” of these species.

Accuracy checking of each individual gene alignment (using nucleotide sequences) was performed by Guidance (Penn et al., 2010) using the Mafft algorithm (Katoh et al., 2002) anchored by codons with default options, followed by the removal of low-accuracy alignment sites. All edited alignments were concatenated by FASconCAT yielding a nucleotide supermatrix (Kück and Meusemann, 2010). The best partitioning scheme and evolutionary model for each partition were calculated by PartitionFinder (Lanfear et al., 2012), which tests all available models under the Bayesian Information Criterion (BIC) selection procedure (Lanfear et al., 2014). Maximum likelihood (ML) analysis for phylogeny construction was performed using IQTree v.1.1.5 assuming the best partitioning and respective models according to the previous step (Nguyen et al., 2015). A total of 1000 bootstrap pseudoreplicates were performed to assess clade support. Additional taxa included to strengthen the confidence in the phylogenetic relationships are as follows: *Xanthomonas fragariae* (XfrLMG25863, RefSeq PRJNA80793: Vandroemme et al., 2013), *Xanthomonas arboricola* pv. *corylina* (XacNCCB100457, RefSeq PRJNA193452: Ibarra Caballero et al., 2013), *Xanthomonas campestris* pv. *musacearum* (XcmNCPB4384, RefSeq PRJNA73881: Wasukira et al., 2012), *Xanthomonas axonopodis* pv. *citrumelo* F1 (XalfaF1, RefSeq PRJNA73179: Jalan et al., 2011), *Xanthomonas oryzae* pv. *oryzae* (XooKACC10331, RefSeq PRJNA12931: Lee et al., 2005), *Xanthomonas campestris* pv. *campestris* (XccATCC33913, RefSeq PRJNA57887: da Silva et al., 2002), *Xanthomonas euvesicatoria* (also *Xanthomonas campestris* pv. *vesicatoria*, Xe85-10, RefSeq PRJNA58321: Thieme et al., 2005).

### Whole genome SNP analysis

Illumina reads were trimmed using Trimmomatic version 0.32 (Bolger et al., 2014) and were then mapped to the reference genome *Xanthomonas axonopodis* pv. *citri* strain 306 (Xac306, NC_003919: da Silva et al., 2002) using bowtie2 version 2.1.0 (Langmead and Salzberg, 2012). The Best Practices guidelines of the Broad Institute for variant calling were followed (https://www.broadinstitute.org/gatk/guide/best-practices). MarkDuplicates from Picard Tools version 1.118 was used to mark duplicate reads. RealignerTargetCreator and IndelRealigner from GenomeAnalysisToolkit (GATK) version 3.3-0 were used to verify reads were aligned properly (McKenna et al., 2010). HaplotypeCaller from GATK was used to discover variants. SNPs were concatenated as previously described (Bart et al., 2012). A ML phylogenetic tree with bootstrap values was created using RAxML version 8.0 (Stamatakis, 2014).

### Effector allele analysis

Effectors were compared within each species at the amino acid sequence level for *Xp* and the nucleotide level for *Xe* and *Xg*, and each distinct allele was assigned a number. Neighbor-joining trees were constructed to visualize differences in effector profiles among strains in each species. Simple genetic distances among strains in their effector profiles were calculated for all pairwise comparisons within each species, such that a difference at one effector between two strains equaled a distance of 1.0 and a difference at five effectors equaled a distance of 5.0. *Xp* calculations included an outgroup profile from Xe85-10. Distance was calculated using GenAlEx 6.501. Distance matrices were exported to MEGA format and trees were constructed in MEGA 6.06 (Tamura et al., 2013).

### Confirmation of the TAL effector AvrHah1 in *Xg*

*Xg* strains were infiltrated into pepper cv. ECW30R at OD_600_ = 0.3 in order to determine if activation of the *Bs3* resistance gene occurs in response to AvrHah1. Negative and positive controls for AvrHah1 in *Xg* are strain 1782 and 04T5, respectively (Schornack et al., 2008). Pictures were taken 48 h post-infiltration (hpi). For Southern blot analysis, 5 μg of *Xg* DNA (extracted as described above) was restriction digested for 2 h with BamHI and run on a 0.7% agarose gel. DNA was transferred overnight to a Hybond-N+ membrane and hybridized overnight with a P^32^-labeled probe for the first 705bp of AvrHah1. The size of the predicted BamHI-digested AvrHah1 fragment is 2964bp.

### Effector deletion

Insertion mutants in *Xp* strains (ΩavrBsT) were constructed using site-directed homologous recombination of a partial fragment linked to a gene for antibiotic resistance. Intragenetic partial fragments (approximately 500 bp) of each targeted gene were PCR amplified and cloned into the pCR2.1 TOPO-vector using the TA cloning method (Invitrogen). Positive clones were confirmed by Sanger sequencing. The plasmids were introduced into competent cells of *Xp* recipient strains by electroporation, and transformed cells were selected for kanamycin resistance (kan^R^). Single homologous recombination events (due to the integration of the TOPO plasmid containing a portion of the respective gene) disrupted the gene of interest (Sugio et al., 2005). Mutations were confirmed by PCR using a primer flanking the upstream region of the targeted gene and the M13 Forward primer (pCR2.1 TOPO internal primer), followed by Sanger sequencing.

Whole gene knockout strains Xe85-10ΔXopQ, Xg153ΔhrcV, Xp4BΔAvrBsT, and Xp4BΔXopQΔAvrBsT were constructed using the suicide vector pLVC18 containing the contiguous 1kb upstream and 1kb downstream fragments flanking the targeted gene (Lindgren et al., 1986). Double homologous recombination events resulting in markerless deletions were confirmed by PCR or southern blot. Gene deletions were complemented back by conjugation of the stable broad host range plasmid pVSP61 (kan^R^) containing the native promoter and the open reading frame of each respective gene.

### Inoculation conditions

*Xanthomonas* strains were grown on nutrient yeast glycerol agar (NYGA) supplemented, as appropriate, with 100 μg/ml rifampicin (wild type and deletion strains) and 25 μg/ml kanamycin. Strains were incubated at 28°C for 48–72 h. Cells were washed from agar plates with 10mM MgCl_2_, and the concentration was adjusted as necessary. For growth assays, leaves were syringe-infiltrated with bacterial suspensions of 10^5^ CFU/mL. For virulence scoring, leaves were syringe infiltrated at 10^8^ CFU/mL and pictures were taken 48 h post-infiltration (hpi) after submerging leaves in water for 10 min to enhance any water-soaked phenotypes. For lesions assays, leaves were syringe infiltrated at 10^4^ CFU/mL and pictures were taken 8–10 days post-infiltration (dpi) after submerging leaves in water for 10 min.

## Results

### Genome submission

Draft genome sequences of 32 *Xp*, 25 *Xe* and 10 *Xg* field strains, respectively, from diseased peppers and tomatoes in the United States were obtained by Illumina sequencing (Table 1). Genome assembly statistics for each strain and average *de novo* assembly statistics for *Xe*, *Xp*, and *Xg* are presented in Supplemental Tables 1 and 2, respectively. Draft genome sequences have been deposited in the National Center for Biotechnology (Supplemental Table 1).

### Core genome phylogenetic analysis identifies a division in the *Xp* population

The core genome for all three species was identified by sequence similarity, yielding 1152 protein-coding gene families, of which 1017 were considered bona fide orthologs; 135 families were discarded as spurious alignments by the program Guidance. The 1017 families were concatenated, yielding a supermatrix of 916,326 sites. The best partitioning scheme chosen was by codon position in which first, second and third positions are set as separated partitions. The best evolutionary models for each partition were respectively GTR+I+G for the first and second partitions, and TVM+I+G for the third partition.

**Figure 1:**
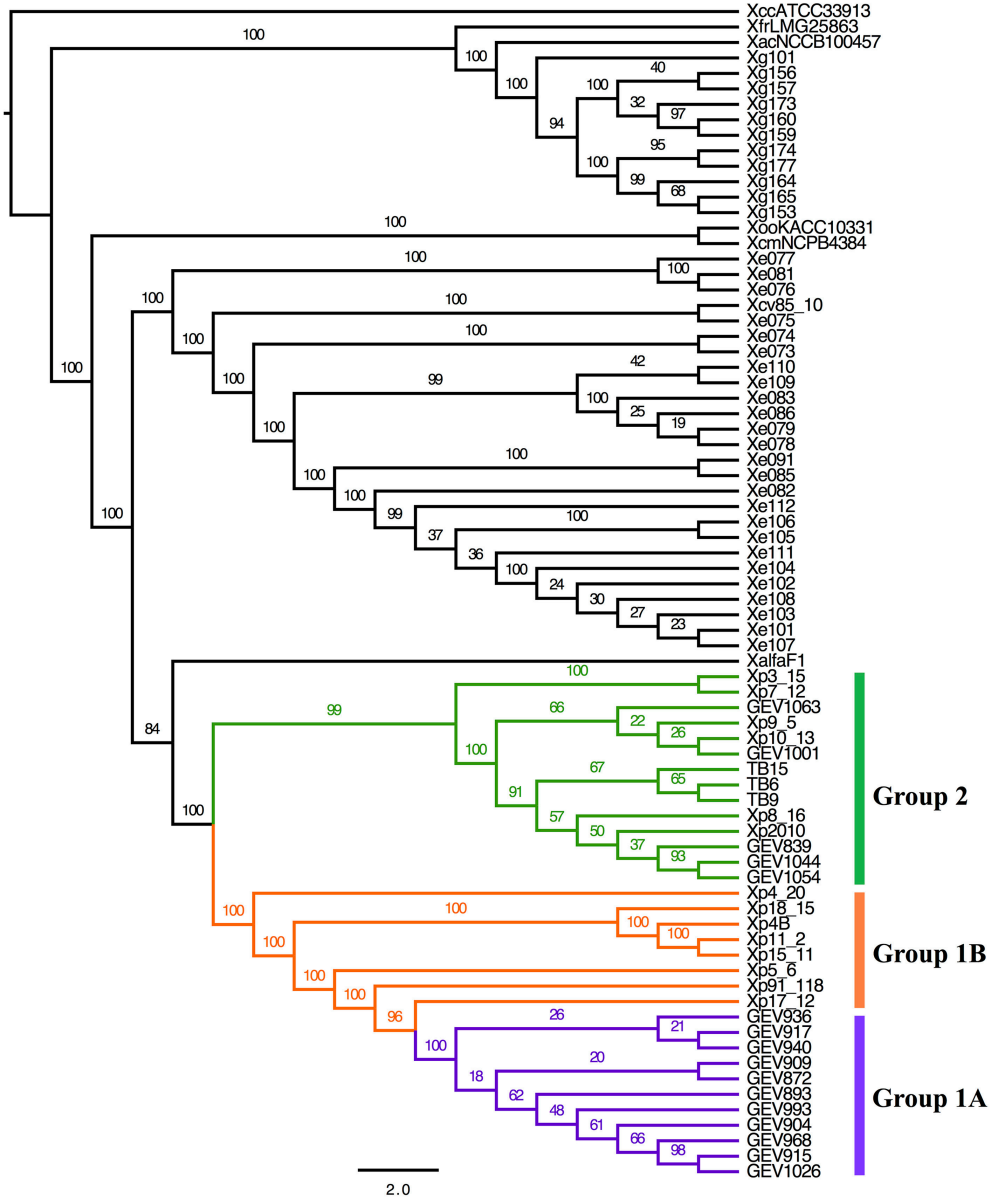
**Core genome phylogenetic analysis**. Phylogenetic trees obtained by ML (IQTree) analysis, based on partitioned analysis (by codon position) of a total of 916,326 sites (1017 genes families). Branch support values are shown for each tree, consisting of relative bootstrap proportions. Brackets to indicate *Xp* group designations are colored as follows: Group 1A, purple; Group 1B, orange; Group 2, green.

The Maximum Likelihood (ML) phylogeny based on core genome orthologs displays *Xe*, *Xp*, and *Xg* behaving as separate monophyletic groups (Figure 1). Our results mirrored previous studies, *Xe* and *Xp* being closely related, and *Xg* more distant phylogenetically, with all three species forming monophyletic groups. For *Xp* strains, this analysis showed a division, which we define here as Group 1—further divided into Group 1A and 1B—and Group 2. Group 1A comprises 11 strains (out of 16) from 2012 that form a monophyletic clade (branches in purple). Other strains belonging to Group 1 are defined here as Group 1B (branches in orange), which includes the reference strain Xp91-118, Xp4B (isolated in 1998), and six strains isolated in 2006. Group 1B does not contain any strains isolated in 2012. We define 14 strains as Group 2 (branches in green) which includes five strains from 2006, the single strain from 2010, five strains from 2012, and all three 2013 strains.

### Whole genome SNP analysis resolves genetic differences among closely related strains

**Figure 2:**
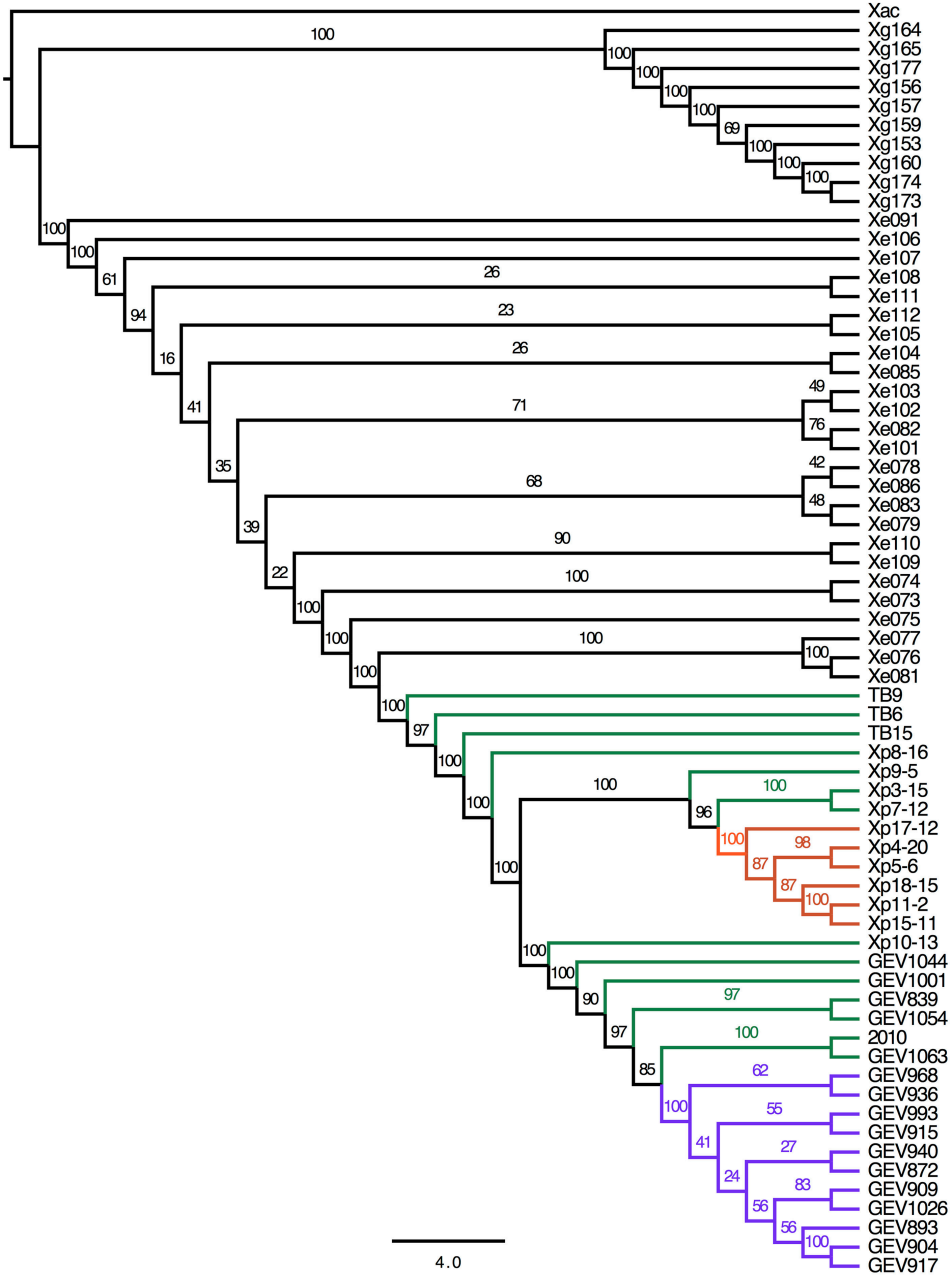
**Phylogeny based on whole genome SNP analysis**. Sequencing reads were mapped to *Xanthomonas axonopodis* pv. *citri* (Xac) reference number NC_003919 and bootstrap values are displayed. Scale bar corresponds to the number of nucleotide substitutions per site. Branches for *Xp* strains are colored to indicate group designations as in Figure 1: Group 1A, purple; Group 1B, orange; Group 2, green.

A total of 225,284 SNPs were identified between the *Xe*, *Xg* and *Xp* genomes compared to the reference Xac306, ranging from 22,105 (Xg164) to 142,272 (GEV1063) (Supplemental Table 3). Average SNPs (± standard deviations) between Xac306 and *Xe*, *Xp* and *Xg* field strains are 128,376 ± 3024, 136,673 ± 3402, and 30,462 ± 8015, respectively. Although the majority of *Xp* strains carry more SNPs between Xac306 than *Xe* strains, two *Xp* field strains (TB6 and TB9) show a number of SNPs within the *Xe* range. SNPs were concatenated and used to build a combined species ML tree (Figure 2). We note that differences in sequencing technology used, genome coverage and large deletions or insertions could potentially skew this analysis and therefore conclusions about branch length between the different species should be avoided. The *Xp* Group 1A clade is retained in the ML SNP phylogeny (branches marked in purple). However, Group 2 (green branches) is interrupted by Group 1B strains (orange branches).

### Effector predictions for *Xanthomonas* field strains identifies differences in effector content compared to reference genomes

Type III effector repertoires from *Xe*, *Xp*, and *Xg* field strains were compared to the appropriate reference strains Xe85-10, Xp91-118, and Xg101 in order to determine if effector repertoires differed between strains with respect to the presence or absence of whole effectors, mutations rendering effectors inactive, or alternate alleles of effectors (Thieme et al., 2005; Potnis et al., 2011).

#### Xe

Several differences were found in the effector content of *Xe* field strains compared to the reference Xe85-10 (Supplemental Table 4). Firstly, Xe85-10 does not have the effector XopAE, which is a translational fusion of the *hrp* cluster members *hpaG* and *hpaF* as seen in Xp91-118 (Potnis et al., 2011). Similar to Xe85-10, field strains isolated before 1997 have separate *hpaG* and *hpaF* genes, whereas *Xe* field strains isolated after 1997 possess the predicted *hpaG*/*hpaF* translational fusion XopAE. Secondly, strains collected after 1997 possess a XopAF-like effector. The effector has 31% amino acid identity to XopAF of Xp91-118, 80% amino acid identity to *X. fuscans* XopAF (WP_022560489.1) and is identical to an effector of *X. citri* pv. *citri* (WP_015472934.1) except for an in-frame internal 12 amino acid deletion. Similarly, the *Xe* strains isolated after 1997 possess XopE3, which shares 97% amino acid identity with XopE3 from *X. arboricola* pv. *pruni* (WP_014125894.1). All field strains of *Xe* but one lack XopG, which is carried by the reference strain Xe85-10. A predicted protein-tyrosine phosphatase (abbreviated PTP) was detected in Xe075 that is not present in any other *Xe* strains. Twelve effectors present in all *Xe* strains isolated between 1985 and 2012 have no nucleotide polymorphisms (Table 2). *Xe* field strains in our collection isolated after 1997 did not contain polymorphisms in *xopAA*, *xopF1*, *xopN*, and *xopO*. Except for Xe85-10 and Xe075, all *Xe* strains have identical sequences for effectors *xopAI*, *xopQ* and *xopV*.

**Table 2:** ***Xanthomonas euvesicatoria* nucleotide type III effector alleles**.

**Type III Effector**	**Year**	**1985**	**1994**		**1995**			**1996**	**1997**	**1998**		**1999**		**2000**	**2003**	**2004**				**2008**		**2009**	**2010**			**2011**	**2012**
	**State**		**NC**	**NC**	**F**	**F**	**F**	**K**	**F**	**F**	**F**	**F**	**F**	**F**	**F**	**G**	**G**	**G**	**G**	**NC**	**F**	**NC**	**NC**	**NC**	**NC**	**NC**	**NC**
	**Strain**	**85-10**	**Xe073**	**Xe074**	**Xe075**	**Xe076**	**Xe081**	**Xe077**	**Xe078**	**Xe079**	**Xe082**	**Xe083**	**Xe085**	**Xe086**	**Xe091**	**Xe109**	**Xe110**	**Xe111**	**Xe112**	**Xe101**	**Xe102**	**Xe103**	**Xe104**	**Xe105**	**Xe106**	**Xe107**	**Xe108**
AvrBs2		4	7	7	7	7	4	7	3	1	2	6	5	6	5	1	1	2	2	2	2	2	2	2	2	2	2
XopA		1	1	1	1	1	1	1	1	1	1	1	1	1	1	1	1	1	1	1	1	1	1	1	1	1	1
XopAA		1	2	2	1	1	1	1	2	2	2	2	2	2	2	2	2	2	2	2	2	2	2	2	2	2	2
XopAD		1	2	2	1	3	3	1	1	1	1	1	1	1	1	1	1	1	1	1	1	1	1	1	1	1	1
XopAE		0	0	0	0	0	0	0	1	1	1	1	1	1	1	1	1	1	1	1	1	1	1	1	1	1	1
XopAF-like		0	1	1	0	0	1	0	1	1	1	1	1	1	1	1	1	1	1	1	1	1	1	1	1	1	1
XopAI		1	2	2	1	2	2	2	2	2	2	2	2	2	2	2	2	2	2	2	2	2	2	2	2	2	2
XopAK		1	1	1	1	1	1	1	1	1	1	1	1	1	1	1	1	1	1	1	1	1	1	1	1	1	1
XopB		1	1	1	1	2	2	2	1	1	1	3	1	1	1	1	1	1	1	1	1	1	1	1	1	1	1
XopC1		1	1	1	1	1	1	1	1	1	1	1	1	1	1	1	1	1	1	1	1	1	1	1	1	1	1
XopD		1	1	1	1	2	2	3	1	1	1	1	1	1	1	1	1	1	1	1	1	1	1	1	1	1	1
XopE1		1	1	1	1	1	1	1	1	1	1	1	1	1	1	1	1	1	1	1	1	1	1	1	1	1	1
XopE2		1	4	3	1	1	1	3	1	1	2	1	1	1	1	1	1	2	2	2	2	2	2	2	2	2	2
XopE3		0	1	1	0	0	1	0	1	1	1	1	1	1	1	1	1	1	1	1	1	1	1	1	1	1	1
XopF1		1	1	1	1	1	1	1	2	2	2	2	2	2	2	2	2	2	2	2	2	2	2	2	2	2	2
XopF2		1	1	1	1	1	1	1	1	1	1	1	1	1	1	1	1	1	1	1	1	1	1	1	1	1	1
XopG		1	0	0	1	0	0	0	0	0	0	0	0	0	0	0	0	0	0	0	0	0	0	0	0	0	0
XopI		1	1	1	1	1	1	1	1	1	1	1	1	1	1	1	1	1	1	1	1	1	1	1	1	1	1
XopJ1		1	1	1	1	1	1	1	1	1	1	1	1	1	1	1	1	1	1	1	1	1	1	1	1	1	1
XopJ3		1	1	1	1	1	1	1	1	1	1	1	1	1	2	1	1	1	1	1	1	1	1	1	1	1	1
XopK		1	1	1	1	1	1	1	1	1	1	1	1	1	1	1	1	1	1	1	1	1	1	1	1	1	1
XopL		1	1	1	1	1	1	1	1	1	1	1	1	1	1	1	1	1	1	1	1	1	1	1	1	1	1
XopN		1	2	2	1	1	1	1	2	2	2	2	2	2	2	2	2	2	2	2	2	2	2	2	2	2	2
XopO		1	2	2	2^CTG^	1^T^	1^T^	1^T^	2	2	2	2	2	2	2	2	3	2	2	2	2	2	2	2	2	2	2
XopP		1	1	1	1	1	1	1	1	1	1	1	1	1	1	1	1	1	1	1	1	1	1	1	1	1	1
XopQ		1	2	2	1	2	2	2	2	2	2	2	2	2	3	2	2	2	2	2	2	2	2	2	2	2	2
XopR		1	1	1	1	1	1	1	1	1	1	1	1	1	1	1	1	1	1	1	1	1	1	1	1	1	1
XopV		1	2	2	1	2	2	2	2	2	2	2	2	2	2	2	2	2	2	2	2	2	2	2	2	2	2
XopX		1	1	1	1	1	1	1	1	1	1	1	1	1	1	1	1	1	1	1	1	1	1	1	1	1	1
XopZ1		1	1	1	1	1	1	1	1	2	1	1	1	1	1	1	1	1	1	1	1	1	1	3	1	1	1
PTP		0	0	0	1	0	0	0	0	0	0	0	0	0	0	0	0	0	0	0	0	0	0	0	0	0	0

*Each distinct nucleotide allele was assigned an arbitrary number. The number 0 indicates the effector is missing from genomic assemblies. Effector PTP, protein tyrosine phosphatase. Superscripts are as follows: T, truncation; CTG, contig break in assembly unable to be confirmed via Sanger Sequencing*.

**Figure 3:**
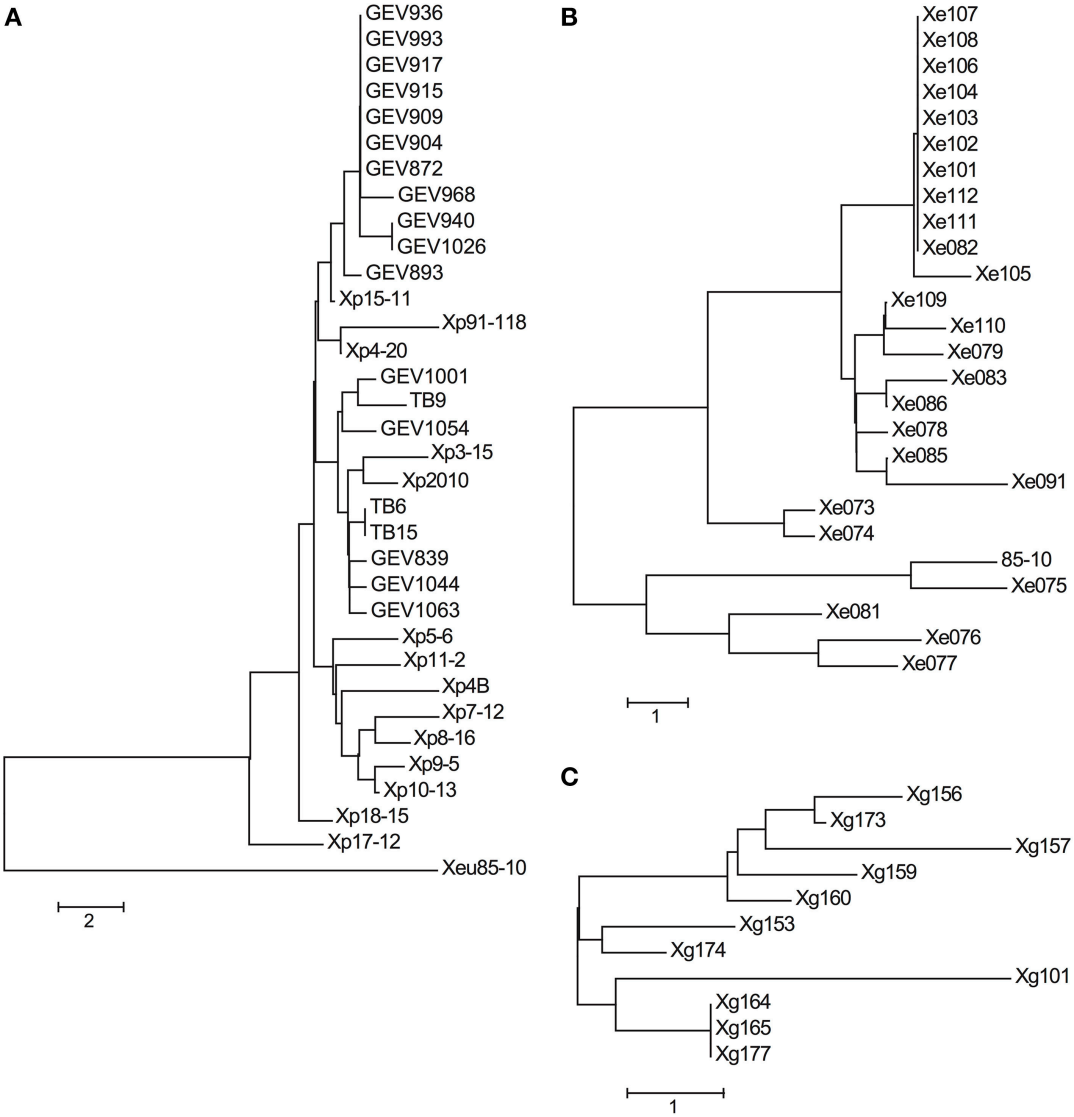
**Neighbor-joining trees of effector allele profiles**. Neighbor-joining trees for *Xp* **(A)**, *Xe* **(B)**, and *Xg* **(C)** field strains were constructed using nucleotide (*Xe* and *Xg*) and amino acid (*Xp*) pairwise allele differences between strains. Effector allele designations can be found in Tables 2–**4**. A difference at one effector between two strains equals a distance of 1.0.

The neighbor-joining tree of the effector alleles displays a grouping of the seven *Xe* strains isolated before 1997, and a clade of 11 strains with nearly identical allele profiles isolated from 2004 to 2012 (Figure 3B). Although Xe111 and Xe112 group with the clade of 11 strains and were isolated in Georgia in 2004, two other Georgia 2004 strains, Xe109 and Xe110, are separated from this clade due to differences in *avrBs2*, *xopE2*, and *xopO*. Interestingly, Xe082 was isolated in 1998 but has an effector allele profile similar to the 11-member clade made up of strains isolated between 2004 and 2012.

#### Xp

A shift in pathogen populations from tomato race 3 to tomato race 4 has been observed in Florida (Horvath et al., 2012). All the strains sequenced here (with isolation years spanning from 1998 to 2013 in Florida) are tomato race 4 strains and contain null mutations in the *xopAF*/*avrXv3* gene of the reference strain Xp91-118 (Supplemental Table 5). All strains possess XopJ4/AvrXv4 with the exception of the pepper strain Xp2010. Another effector, AvrBsT, which has been associated with hypersensitive response (HR) on pepper (Minsavage et al., 1990), has not been previously reported in *Xp*. Xp4B, which was isolated in 1998, has AvrBsT and is non-pathogenic on pepper (Supplemental Figures 2, 3). AvrBsT is also present in nine strains (out of 11) that were isolated in 2006, in all 16 strains collected in 2012, and in one of the three strains collected in 2013. Interestingly, strain Xp17-12 (isolated in 2006) contains two effectors, XopF2 and XopV, that have sequences identical to the corresponding Xe85-10 effector sequence (Table 3). Effectors XopD and XopAD exhibit different alleles in the strains isolated in 2012. All strains have XopE2, which was absent in the reference strain Xp91-118. XopE2 is also present in all *Xe* and *Xg* field and reference strains. A subset of the *Xp* 2006 population have XopE4, which had been reported only in *X. fuscans* pv. *aurantifolii* (Moreira et al., 2010). However, XopE4 is not present in any strains from 2010, 2012, or 2013. Interestingly, strains belonging to *Xp* Group 2 possess a XopQ identical to the allele from Xe85-10. The neighbor-joining tree based on effector alleles shows the conservation of Group 1A, but Group 1B and Group 2 strains were intertwined (Figure 3A).

**Table 3:** ***Xanthomonas perforans* amino acid type III effector alleles**.

**Type III effector**	**Year**	**1991**	**1998**	**2006**											**2010**	**2012**																**2013**			**1985**
	**State**	**F**	**F**	**G**	**G**	**G**	**F**	**F**	**F**	**F**	**F**	**F**	**F**	**F**	**F**	**F**	**F**	**F**	**F**	**F**	**F**	**F**	**F**	**F**	**F**	**F**	**F**	**F**	**F**	**F**	**F**	**F**	**F**	**F**	**F**
	**Strain**	**Xp91-118**	**Xp4B**	**Xp3-15**	**Xp4-20**	**Xp5-6**	**Xp7-12**	**Xp8-16**	**Xp9-5**	**Xp10-13**	**Xp11-2**	**Xp15-11**	Xp17-12^*^	**Xp18-15**	Xp2010^*^	**GEV839**	**GEV872**	**GEV893**	**GEV904**	**GEV909**	**GEV915**	**GEV917**	**GEV936**	**GEV940**	**GEV968**	**GEV993**	**GEV1001**	**GEV1026**	**GEV1044**	**GEV1054**	**GEV1063**	**TB6**	TB9^*^	TB15^*^	**Xe 85-10**
AvrBs2		1	1	1	1	1	1	1	1	1	1	1	1	1	1	1	1	1	1	1	1	1	1	1	1	1	1	1	1	1	1	1	1	1	2
XopA		1	1	1	1	1	1	1	1	1	1	1	1	1	1	1	1	1	1	1	1	1	1	1	1	1	1	1	1	1	1	1	1	1	2
XopAP		1	1	1	1	1	1	1	1	1	1	1	1	1	1	1	1	1	1	1	1	1	1	1	1	1	1	1	1	1	1	1	1	1	2
XopAR		1	1	1	1	1	1	1	1	1	1	1	1	1	1	1	1	1	1	1	1	1	1	1	1	1	1	1	1	1	1	1	1	1	0
XopC2		1	1	1	1	1	1	1	1	1	1	1	1	1	1	1	1	1	1	1	1	1	1	1	1	1	1	1	1	1	1	1	1	1	2
XopE1		1	1	1	1	1	1	1	1	1	1	1	1	1	1	1	1	1	1	1	1	1	1	1	1	1	1	1	1	1	1	1	3	1	2
XopF1		1	1	1	1	1	1	1	1	1	1	1	1	1	1	1	1	1	1	1	1	1	1	1	1	1	1	1	1	1	1	1	1	1	2
XopF2		1	1	1	1	1	1	1	1	1	1	1	2	1	1	1	1	1	1	1	1	1	1	1	1	1	1	1	1	1	1	1	1	1	2
XopI		1	1	1	1	1	1	1	1	1	1	1	1	1	1	1	1	1	1	1	1	1	1	1	1	1	1	1	1	1	1	1	1	1	2
XopJ4		1	1	2	1	1	2	1	1	1	1	1	1	1	0	1	1	1	1	1	1	1	1	1	1	1	1	1	1	1	1	1	1	1	0
XopK		1	1	1	1	1	1	1	1	1	1	1	1	1	1	1	1	1	1	1	1	1	1	1	1	1	1	1	1	1	1	1	1	1	2
XopL		1	1	1	1	1	1	1	1	1	1	1	1	1	1	1	1	1	1	1	1	1	1	1	1	1	1	1	1	1	1	1	1	1	2
XopN		1	1	1	1	1	1	1	1	1	1	1	2	1	1	1	1	1	1	1	1	1	1	1	1	1	1	1	1	1	1	1	1	1	3
XopQ		1	1	2	1	1	2	2	2	2	1	1	1	1	2	2	1	1	1	1	1	1	1	1	1	1	2	1	2	2	2	2	2	2	2
XopR		1	1	1	1	1	1	1	1	1	1	1	1	1	1	1	1	1	1	1	1	1	1	1	1	1	1	1	1	1	1	1	1	1	2
XopV		1	1	1	1	1	1	1	1	1	1	1	2	1	1	1	1	1	1	1	1	1	1	1	3	1	1	1	1	1	1	1	1	1	4
XopX		1	1	1	1	1	1	1	1	1	1	1	1	1	1	1	1	1	1	1	1	1	1	1	1	1	1	1	1	1	1	1	1	1	2
XopAK		1	1	1	1	1	1	1	1	1	1	1	1	1	1	1	1	1	1	1	1	1	1	1	1	1	1	1	1	1	1	1	1	1	2
XopD		3	3	3	3	3	3	3	3	3	3	3	3	3	3	3	4	5	4	4	4	4	4	4	4	4	7	4	3	6	3	3	7	3	8
XopAD		1	1	1	1	1	1	1	1	1	1	1	3	3	1	1	1	1	1	1	1	1	1	2	1	1	1	2	1	1	1	1	1	1	3
XopE2		0	2	2	2	2	2	2	2	2	2	2	2	2	2	2	2	2	2	2	2	2	2	2	2	2	2	2	2	2	2	2	2	2	1
XopE4		0	0	1	0	1	0	0	0	0	0	0	0	0	0	0	0	0	0	0	0	0	0	0	0	0	0	0	0	0	0	0	0	0	0
AvrBsT		0	1	1	1	0	1	1	1	1	1	1	0	1	0	1	1	1	1	1	1	1	1	1	1	1	1	1	1	1	1	1	0	0	0
XopP1		1	1	1	1	0	3	4	0	0	5	1	1	1	1	1	1	1	1	1	1	1	1	1	1	1	1	1	1	1	1	1	1	1	0
XopP2		2	5	17	2	4	8	6	9	0	3	1	1	18	15	14	1	1	1	1	1	1	1	1	1	1	10	1	11	12	13	19	19	19	16
XopP3		0	0	0	0	0	1	1	1	0	2	0	0	0	0	0	0	0	0	0	0	0	0	0	0	0	0	0	0	0	0	0	0	0	0
XopZ1		1	1	1	1	1	1	1	1	1	1	1	1	1	1	1	1	1	1	1	1	1	1	1	1	1	1	1	1	1	1	1	1	1	2
XopAE		1	1	1	1	1	1	1	1	1	1	1	1	1	1	1	1	1	1	1	1	1	1	1	1	1	1	1	1	1	1	1	1	1	2
XopAF		1	0	0	0	0	0	0	0	0	0	0	0	0	0	0	0	0	0	0	0	0	0	0	0	0	0	0	0	0	0	0	0	0	0
Group		1B	1B	2	1B	1B	2	2	2	2	1B	1B	1B	1B	2	2	1A	1A	1A	1A	1A	1A	1A	1A	1A	1A	2	1A	2	2	2	2	2	2	

*Each distinct amino acid allele was assigned an arbitrary number. The number 0 indicates the effector is missing from genomic assemblies. AvrBsT and XopP were not used to make the Neighbor-joining effector allele trees for Xp in Figure 3A. Asterisk (*.

* *) indicates newly identified Xp pepper pathogens*.

#### Xg

The collection of *Xg* field isolates spans 3 years and covers two states (Ohio and Michigan). Effector predictions in *Xg* field strains from this period revealed the presence of four potential effectors that are not present in the reference strain Xg101, which was isolated in the southeastern Europe in 1953 (Supplemental Table 6). *Xg* field strains possess a XopJ1 that is identical to the allele in Xe85-10 and a type III effector protein (T3EP) that has 78% amino acid identity to a predicted *Ralstonia* peptidase effector (WP_014619440.1). A predicted effector of the *Xg* strains shares 65% amino acid similarity to a *X. campestris* pv. *campestris* PTP type III effector (WP_011345706.1). Two copies of XopE2 are present in 7 out of 10 *Xg* field strains, while the remaining three and the reference strain Xg101 have only one XopE2. Two field strains carry the effector AvrBs7 (Potnis et al., 2012). Because the repetitive nature of TAL effector genes renders them difficult to assemble from short reads, Southern blot analysis was used to identify potential family members (Supplemental Figure 3A). In addition, the ability of each strain to induce a HR on pepper cv. ECW30R, which contains the cognate R gene *Bs3* to the TAL effector AvrHah1 was tested (Supplemental Figure 3B). All field strains of *Xg* contained a single TAL effector, an apparent AvrHah1, on the basis of band size and activity.

Although the *Xg* strains were isolated within a 3-year period, only three *Xg* field strains (Xg164, Xg165, and Xg167) have identical effector allele profiles at the nucleotide level (Figure 3C). Three effectors are highly polymorphic: the *avrBs1*-class effector, of which three alleles were detected, and the two *xopE2* effectors, of which five and three alleles were detected (Table 4). Two alleles of *xopAD* are present at equal frequencies in the *Xg* field strains, with both alleles present in field strains isolated in the same year in the same state (e.g., Xg165 and Xg173, Ohio 2011) and in the same year in different states (e.g., Xg153 and Xg156, Ohio and Michigan, respectively, 2010).

**Table 4:** ***Xanthomonas gardneri* nucleotide type III effector alleles**.

**Type III effector**	**Year**	**1953**	**2010**					**2011**			**2012**	
	**State**		**O**	**M**	**M**	**M**	**M**	**O**	**O**	**O**	**O**	**O**
	**Strain**	**Xg101**	**Xg153**	**Xg156**	**Xg157**	**Xg159**	**Xg160**	**Xg164**	**Xg165**	**Xg173**	**Xg174**	**Xg177**
AvrBs1 class		1	2	3	3	3	3	2	2	3	2	2
AvrBs2		1	1	1	1	1	1	1	1	1	1	1
AvrBs7		0	0	1	0	0	0	0	0	1	0	0
AvrHah1		na	na	na	na	na	na	na	na	na	na	na
AvrXccA1		1	1	1	1	1	1	1	1	1	1	1
XopAD		1	1	2	2	2	2	1	1	2	1	1
XopAM		1	1	1	1	1	1	1	1	1	1	1
XopAO		1	1	1	1	1	1	1	1	1	1	1
XopAQ		1	1	0	0	1	1	1	1	1	1	1
XopAS		1	1	1	1	1	1	1	1	1	1	1
XopB		1	1	1	1	1	1	1	1	1	1	1
XopD		1	1	1	1	1	1	1	1	1	1	1
XopE2_0		1	1	4	5	3	1	5	5	4	2	5
XopE2_1		0	1	1	3	2	1	0	0	1	1	0
XopF1		1	1	1	1	1	1	1	1	1	1	1
XopG		1	FS	1	1	1	1	1	1	1	1	1
XopJ1		0	1	1	1	1	1	1	1	1	1	1
XopK		1	1	1	1	1	1	1	1	1	1	1
XopL		1	1	1	1	1	1	1	1	1	1	1
XopN		1	1	1	1	1	1	1	1	1	1	1
XopQ		1	1	1	1	1	1	1	1	1	1	1
XopR		1	1	1	1	1	1	1	1	1	1	1
XopX		1	1	1	1	1	1	1	1	1	1	1
ZopZ2		1	1	1	2	1	1	1	1^CTG^	1	1	1
PTP		0	1	1	1	1	1	1	1	1	1	1
T3EP		0	1	1	1	1	1	1	1	1	1	1

*Each distinct nucleotide allele was assigned an arbitrary number. The number 0 indicates the effector is missing from genomic assemblies. PTP, protein tyrosine phosphatase; T3EP, type III effector protein. The TAL effector AvrHah1 could not be assembled (na, not assembled). Superscripts are as follows: CTG, contig break in assembly unable to be confirmed via Sanger Sequencing; FS, a frame shift mutation*.

#### Common effectors between species

Effector predictions of the field strains has identified two new common putative effectors to add to the previously described list of 11 effectors shared between *Xe*, *Xp*, and *Xg* (Potnis et al., 2011). XopE2 was identified in all *Xp* field strains and, while not in the reference Xp91-118, should, therefore, be considered a commonly shared effector with *Xe* and *Xg*. The identification of AvrBsT in the majority of *Xp* field strains and an identical copy of *Xe* XopJ1 in *Xg* field strains indicates the presence of a more broadly defined YopJ-family effector to the commonly shared effector list.

### Association of AvrBsT presence or absence in host range expansion of *Xp* on pepper

*Xp* has previously been considered restricted to tomato as a host. In 2010, we isolated a strain from a greenhouse-grown diseased pepper plant. This strain was confirmed as *X. perforans* based on 16S rRNA sequencing and multilocus sequence analysis (MLSA) (Timilsina et al., 2015), and is designated here as strain Xp2010. Xp2010 does not induce a hypersensitive response (HR) on pepper cv. Early CalWonder (ECW) and is able to create foliar disease lesions (Supplemental Figure 1). Effector predictions for Xp2010 indicated that the absence of AvrBsT, which induces HR on pepper (Kim et al., 2010), may be responsible for its pepper host expansion. We were curious to see if other *Xp* strains in our collection displayed host expansions to pepper similar to Xp2010 and if this could be explained solely by the absence of AvrBsT. We used PCR to confirm the effector prediction results for the presence or absence of AvrBsT in the *Xp* field strains and inoculated pepper cv. ECW with a high inoculum (10^8^ CFU/ml) to determine which strains induce HR (Supplemental Table 7). We confirmed that four additional field strains, Xp5-6, Xp17-12, TB9, and TB15 do not possess AvrBsT and also fail to induce HR. Xp17-12, TB9, and TB15 but not Xp5-6 are able to cause disease lesions on pepper cv. ECW when infiltrated at a low inoculum (10^4^ CFU/ml) (Supplemental Figure 7), indicating that additional factors restrict the host range of Xp5-6 on pepper.

**Figure 4:**
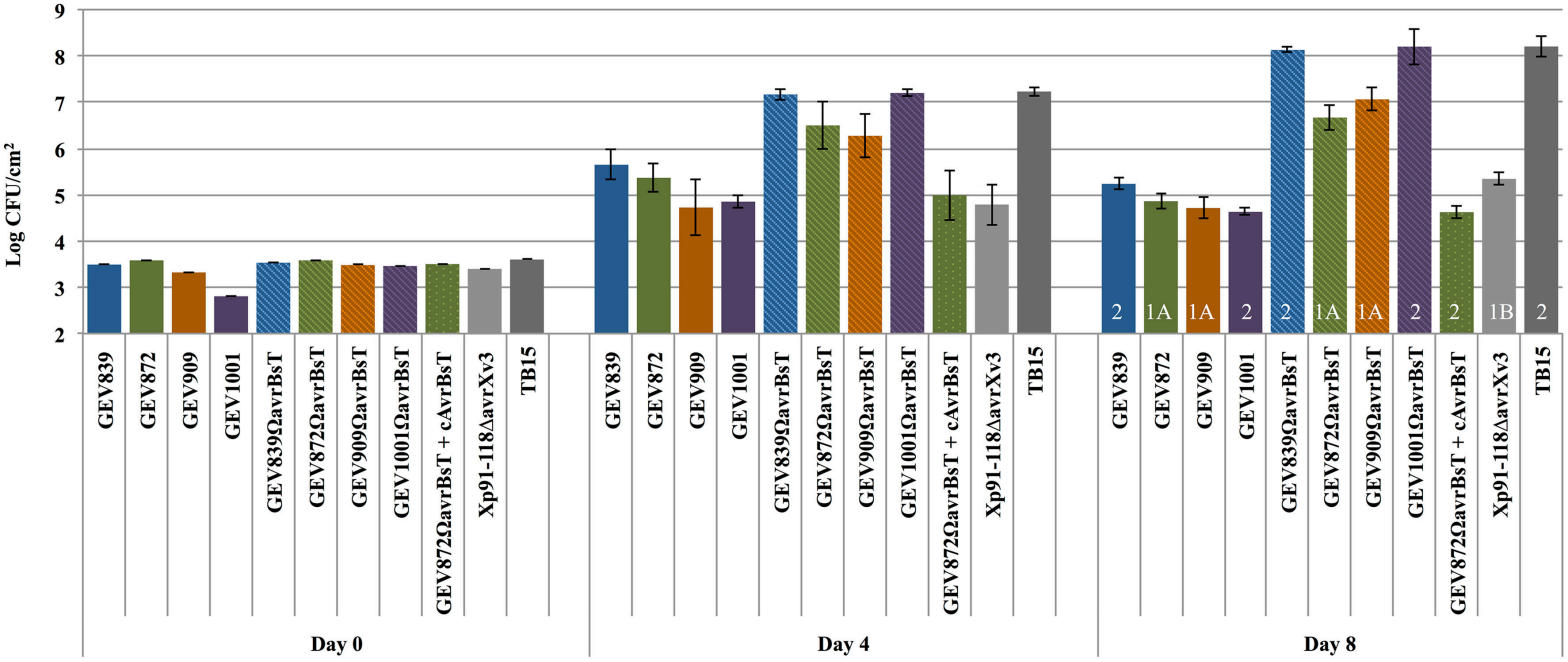
**Role of avrBsT as host range determinant on pepper cv. Early CalWonder**. *In planta* growth of *X. perforans* strains and *avrBsT* insertion mutants was measured at different time points (days 0, 4, and 8) after infiltration of leaves of pepper cv. Early CalWonder (ECW) using an inoculum concentration of 10^5^ CFU/ml. Group designations are marked in white over Day 8 growth.

Three of the newly identified pepper pathogens (Xp2010, TB9, and TB15) belong to Group 2. We observed no HR but differences in pathogenicity and lesion development for the two Group 1B strains that lack AvrBsT (Xp5-6 and Xp17-12). Strain Xp5-6 showed a phenotype similar to Xp91-118Δ*avrXv3*, which is unable to cause lesions on pepper (Supplemental Figure 1). We hypothesized that Group 2 strains carrying mutations in AvrBsT would exhibit *in planta* growth and virulence similar to that of virulent strains from pepper in our collection. At the same time, strains belonging to Group 1 and carrying mutations in AvrBsT would be non-pathogenic on pepper, similar to Xp91-118 Δ*avrXv3* (Astua-Monge et al., 2000). To test this hypothesis, AvrBsT insertion mutants were introduced into two Group 2 strains, GEV839 and GEV1001, and two Group 1A strains, GEV872 and GEV909. Indeed, XpGEV839Ω*avrBsT* and XpGEV1001Ω*avrBsT* from Group 2 lose ability to elicit HR in pepper and are virulent similar to TB15 (Figure 4). *In planta* population levels for these two mutants were not significantly different from TB15 at Days 4 and 8 post-infiltration, indicating that AvrBsT is the lone factor restricting these two strains on pepper. Also as predicted, insertion mutants of *avrBsT* in Group 1A strains GEV872 and GEV909 lose the ability to induce HR on pepper but do not grow to the same extent as TB15. *In planta* populations of XpGEV872Ω*avrBsT* and XpGEV909Ω*avrBsT* were 100-fold higher compared to 91-118Δ*avrXv3* but 20–50-fold lower compared to pepper pathogens XpGEV839Ω*avrBsT*, XpGEV1001Ω*avrBsT* and TB15, indicating the existence of additional factors restricting the virulence of Group 1A strains on pepper.

### Loss of XopQ and AvrBsT expands the host range of *Xp* to *Nicotiana benthamiana*

**Figure 5:**
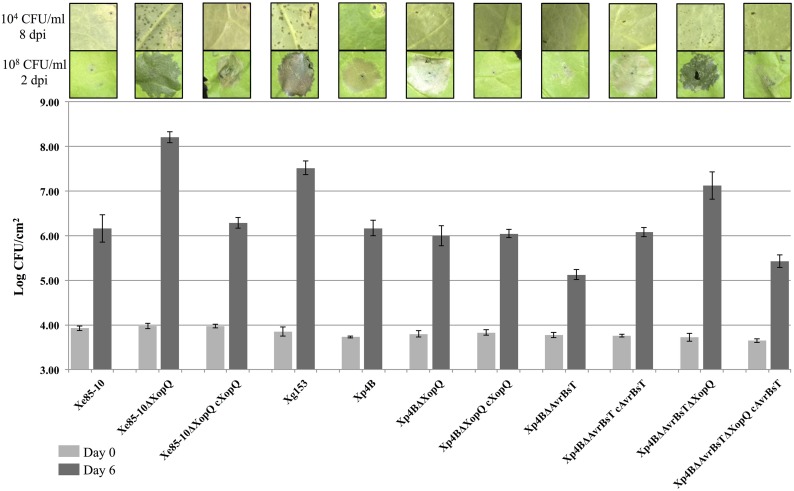
**Host expansion of *Xanthomonas* spp. on *Nicotiana benthamiana***. *In planta* growth of *X. perforans* and AvrBsT and XopQ deletion mutants measured at Days 0 and 6 with a starting inoculum of 10^5^ CFU/mL after infiltration of leaves of *Nicotiana benthamiana*. Infiltrations on *N. benthamiana* were performed at 10^4^ CFU/ml to display lesions and photographed 8 days post-infiltration (8 dpi). High inoculum infiltrated spots were performed at 10^8^ CFU/ml to show HR or water soaking and photographed 2 dpi. This experiment was repeated three times with similar results.

Members of both the XopQ and AvrBsT effector families are known to induce a HR in *N. benthamiana* (Wei et al., 2007; Kim et al., 2010). Family members of XopQ occur in *Xe*, *Xp* and *Xg*. It has previously been shown that a *Pseudomonas syringae* pv. *tomato* DC3000 mutant deficient for the XopQ homolog HopQ1-1 causes disease in *N. benthamiana* (Wei et al., 2007). Xe85-10 is not pathogenic on *N. benthamiana*, causing a weak HR (Figure 5, Xe85-10ΔXopQ). A deletion of XopQ in strain Xe85-10 results in a strain that causes water soaking, disease lesions, and grows to a high titer after 6 days on *N. benthamiana* (Figure 5, Xe85-10ΔXopQ). Complementation of the deletion with plasmid pVSP61 carrying the Xe85-10 allele of XopQ restored the original phenotype of low virulence and enhanced the HR phenotype of the complemented strain (Figure 5, Xe85-10ΔXopQ cXopQ).

All *Xp* field strains contain XopQ and the majority of *Xp* strains contain AvrBsT. Therefore, *Xp* derivative strains in Xp4B were constructed with single gene deletions of XopQ and AvrBsT, and deletions of both XopQ and AvrBsT. Single knockouts for XopQ and AvrBsT in Xp4B remained incompatible on *N. benthamiana* (HR, low growth, no lesions, Figure 5), although Xp4BΔAvrBsT experienced reduced growth compared to Xp4B and Xp4BΔXopQ that was complemented back by the addition of AvrBsT. The double effector deletion mutant Xp4BΔXopQΔAvrBsT gave disease lesions at a low inoculum, showed water soaking at a high inoculum, and grew to levels comparable with Xe85-10ΔXopQ and Xg153 after 6 days on *N. benthamiana* (Figure 5). Consistent with the low virulence gain on pepper by Group 1A AvrBsT mutants, Xp4BΔAvrBsT was able to induce weak lesions on pepper cv. ECW (Supplemental Figure 1), but did not grow to comparable population levels of pepper pathogens Xe85-10 or Xg153 (Supplemental Figure 2).

## Discussion

The population dynamics of *Xanthomonas*-infecting pepper and tomato has shifted in the United States over the past 25 years. Prior to 1991, *Xe* was the prevalent species and the only species in tomato fields in Florida. *Xp* tomato race 3 was identified first in 1991 and eventually replaced the *Xe* population in tomato fields, a process attributed to the ability of *Xp* race 3 to produce bacteriocins against *Xe* strains (Tudor-Nelson et al., 2003; Hert et al., 2005). Xp4B, a tomato race 4 strain identified in 1998, carries a mutation in the *avrXv3* gene. Field surveys thereafter in 2006 and 2012 recovered a majority of race 4 strains carrying either frameshift mutations or transposon insertions in *avrXv3* (Horvath et al., 2012). The first reports of *Xg* in the United States occurred in Ohio and Michigan during a bacterial spot outbreak on tomato in 2009 (Ma et al., 2011).

Here, we sequenced *Xe, Xp, and Xg* strains isolated in different years, from different fields/transplant houses throughout southeastern and midwestern United States. We have also sequenced strains collected during the same season from the same field. Following typical population genomic studies, we have taken three components into consideration; location, time and niche (Monteil et al., 2013). Combining genomic data with metadata such as plant host source, year and location of isolation provides inference of population structure and clues to host adaptation. We have computationally predicted the type III effector repertories for each strain, and have used two different methods in order to infer evolutionary relationships of strains based on whole genome data. Phylogeny based on the core genome considers orthologous genes among the set of genomes considered. Phylogeny based on whole genome SNPs included core as well as variable regions of the genome, and thus provides an additional method to describe the genetic diversity within field strains. Phenotypic data, in particular, host range, was then correlated with the whole genome phylogenies.

MLSA studies showed the presence of two distinct groups of *Xp* populations that appeared to be clonal within the lineage (Timilsina et al., 2015). However, these studies were based on 6 genes out of 5000 genes. In our study, core ortholog gene phylogeny also revealed two distinct groups among *Xp* populations (Groups 1 and 2), although we were able to further separate Group 1 into Group 1A and 1B. Group 1A contains 11 strains isolated in 2012, whereas Group 1B contains six strains isolated in 2006 in addition to Xp4B and the reference strain Xp91-118, isolated in 1998 and 1991, respectively. Group 2 comprises five strains from 2006, the single strain from 2010, five strains from 2012, and all three 2013 strains. Additionally, we detected genetic diversity among strains that appeared to be clonal from MLSA in previous work (Timilsina et al., 2015), particularly evident in *Xp* Group 1A. In our study, the *Xp* 2006 population was more diverse than the 2012 population, possibly due to the fact that sampling in 2006 was carried out in a broader geographic range in Florida and Georgia. The diversity within the 2006 population is evident from the core genome and SNP phylogenies.

This study re-emphasizes the role of population genomics for identification of elements involved in host-pathogen arms race. The data revealed the emergence of tomato race 4 strains of *Xp* carrying mutations (either frameshift/transposon insertion) in *avrXv3*. Strain Xp91-118 isolated in 1991 was non-pathogenic on pepper even when mutated in *avrXv3* (Astua-Monge et al., 2000), indicating the existence of other factors that restrict its host range on pepper. The majority of *Xp* strains in our collection, isolated after 1998, have acquired AvrBsT, an avirulence protein responsible for restricting host range on pepper. AvrBsT has been shown to be a virulence factor by suppressing defense responses in tomato (Kim et al., 2010), possibly conferring a competitive advantage to pathogens in tomato fields. Four of the five *Xp* strains isolated after 1998 that do not possess AvrBsT are pathogenic on pepper. Interestingly, mutation in *avrBsT* results in differences in the *in planta* populations in pepper when compared between Group 1A and Group 2. *avrBsT* mutants in Group 2 experience a full virulence gain on pepper, whereas *avrBsT* mutants in Group 1A acquire only a partial growth benefit, indicating that additional factors restrict the host expansion of Group 1A strains onto pepper. Phenotypic characterization, including pepper pathogenicity tests of *avrBsT* mutants, will need to be conducted on other strains in Groups 1 and 2 to support more definitive conclusions.

At the whole genome level, horizontal gene transfer (HGT) of genes that determine phenotypic differences might have occurred frequently enough during evolution to explain the differing degree of pepper pathogenicity between strains belonging to Group 1B. Two *Xp* strains in Group 1B, Xp17-12, and Xp5-6, do not have AvrBsT and do not induce HR on pepper cv. ECW. However, Xp17-12 is able to induce water-soaked disease lesions on pepper when infiltrated into pepper leaves at a low inoculum (10^4^ CFU/mL) whereas Xp5-6 induces only weak lesions. Similar to Xp5-6, an *avrBsT* deletion in the Group 1B strain Xp4B (Xp4BΔAvrBsT) induces weak disease lesions on pepper and acquires only a partial *in planta* growth increase. Incongruence in degree of pathogenicity and clade could partly be due to the loss or gain of effectors through HGT. Xp17-12 contains effector alleles for XopF2 and XopAD that match those found in Xe85-10 but not those of any other *Xp* strain analyzed here, suggesting the occurrence of HGT events that may have contributed to its ability to infect pepper. Xp5-6 does not share any common effector alleles with Xe85-10. Interestingly, all Group 2 strains contain a XopQ allele identical to XopQ in Xe85-10, while *Xp* strains in Group 1 have a different allele. Previous MLSA analysis also showed evidence for recombination events resulting in haplotypes for two housekeeping genes (*gapA* and *gyrB*) in *Xp* Group 2 strains identical to that found in Xe85-10 (Timilsina et al., 2015). Because mutation in *avrBsT* in the tested Group 2 strains results in complete virulence on pepper, Group 2 strains may have emerged from populations that underwent recombination with an Xe85-10-related strain, acquiring new virulence genes for pepper pathogenicity. Homologous recombination between chromosomal DNA of different *Xanthomonas* species by conjugation *in planta* has been previously observed (Basim et al., 1999), while HGT of virulence-associated genes between different lineages within *X. axonopodis* strains has contributed to host range (Mhedbi-Hajri et al., 2013).

Field strain genomic analysis presents an efficient method for deriving the diversity of type III effector repertories. Knowledge of the effector load in the population will inform strategies for achieving broad durable resistance strategies based on R gene deployment. Within each species, we identified several differences in the effector repertoires of *Xe*, *Xp*, and *Xg* field strains, including the gain or loss of effector genes, null mutations, and the presence of alternate alleles. We predicted three effector additions to the overall *Xe* field strain repertoire (XopE3, XopAF-like, and XopAE) and one removal (XopG) in comparison to the reference strain Xe85-10. The most polymorphic effector in *Xe* is *avrBs2*, a phenomenon perhaps explained by the selective pressures of the pepper *Bs2* resistance gene deployed in the early 1990's. Several of the previously reported mutations in *avrBs2* are represented here, with no novel polymorphisms detected (Swords et al., 1996; Wichmann et al., 2005). Generally, the effector predictions for *Xe* field strains isolated between 1994 and 2004 show increased effector polymorphisms compared to strains isolated between 2004 and 2012, indicating that the effector repertoires have stabilized over time in our sampling population. *Xp* field strains have evolved their repertoires by losing/gaining effectors (XopE2, XopE4, AvrBsT), through allelic exchange (as seen with XopQ in Group 2 strains) and by frameshift mutations/transposon insertions (in *avrXv3*). Diversity in effector repertoires is seen even in strains collected from the same field during a single growing season. Strains TB6 and TB15 possess identical type III effector profiles and appear clonal based on core genome phylogeny except for the absence of AvrBsT in TB15. However, this difference has expanded the host range of TB15 to include pepper while TB6 is restricted to tomato. Similar to TB15, TB9 does not possess AvrBsT but has different alleles of XopD and XopE1 compared to TB6 and TB15. We predicted four additions to the *Xg* field strain effector repertoire including a second copy of XopE2 and a XopJ1 identical to *Xe* strains. We also detected allele differences in an AvrBs1-like effector, XopAD, and XopE2. Through this analysis two additional effectors can be added to the previous list of 11 commonly shared effectors between *Xe*, *Xp*, and *Xg* (Potnis et al., 2011): XopE2 and a YopJ-family member (AvrBsT in *Xp*, XopJ1 in *Xg* and *Xe*).

In addition to strain-level variation, allelic diversification in type III effectors was observed at the species level across *Xe*, *Xp*, and *Xg*. Because type III effector repertoires are proposed to be a major factor determining host range (Hajri et al., 2009), it is important to understand the diversity of effectors present in different species that infect common hosts. Although *Xe*, *Xp*, and *Xg* share thirteen core effectors, effector alleles between these three species may be considerably different. For example, the effector AvrBs2 protein sequence shares 99% identity between reference strains Xp91-118 and Xe85-10, but 77% identity to the AvrBs2 in Xg101. Similarly, the XopQ alleles of Xp91-118 and Xe85-10 share 99% identity at the amino acid level, but 58% identity to XopQ from Xg101. Sampling of a genetically diverse population can be informative to reveal the dominant effector alleles in a specific geographical region, which would be the most appropriate alleles to screen for R protein resistance strategies.

Curiously, we discovered a spectrum of host expansion for *Nicotiana benthamiana* involving the effectors XopQ and AvrBsT. While wild type *Xg* is virulent on *N. benthamiana*, a XopQ deletion in Xe85-10 (Xe85-10ΔXopQ) and a double deletion of XopQ and AvrBsT in Xp4B (Xp4BΔXopQΔAvrBsT) results in a *N. benthamiana* host gain. Reducted *in planta* growth of Xp4BΔAvrBsT compared to Xp4B and Xp4BΔXopQ indicates that AvrBsT may play an important virulence role in *N. benthamiana*. Because the XopQ alleles in *Xe* and *Xp* are relatively similar and stable over time in field strains, the potential R protein “R-XopQ” in *N. benthamiana* would be a promising candidate as a resistance tool against *Xe* and *Xp* in pepper and tomato.

The increased speed and dropping cost of DNA sequencing technology combined with the use of genome editing techniques are providing new opportunities for designing resistance strategies against specific pathogens in various crop species. The spread of agricultural pathogens into new niches, either by increasing global movement of food or the emergence of new niches from climate change, makes the continued genomic surveillance of agricultural pathogens a top priority for food security and resistance strategies. Of particular importance are tracking shifts in dominant species and changes in effector repertoires and alleles. Effector maintenance and stability is a key consideration for the future design of durable resistance strategies using R-gene employment into crops.

## Author contributions

JJ, GV, and BS conceived the project. JJ, FW, BS oversaw genomic sequencing. JJ and GV provided Xp strains, DR provided *Xe* strains, SM provided *Xg* strains. GM and AS prepared genomic DNA for sequencing. AS, NP, AA, FW, RB, and JB oversaw genome assemblies. ST helped with *Xp* genome assembly and initial phenotypic characterization of *Xp* strains. NP and AS constructed mutants and tested them phenotypically and for *in planta* growth. GM and DD helped with cloning and constructing mutants. NP, AS, FW, JB, RB, JJ, and BS performed data analyses and interpreted them. NP and AS did effector analyses and EG performed phylogenetic analysis based on effector profiles. Core genome phylogenies were constructed and interpreted by JP, JM, NA, and JS. MW and RB created the whole genome SNP dataset and constructed phylogenies. JM and JP submitted genome sequences to NCBI GenBank. AS and NP wrote final manuscript. All authors approved the final manuscript.

## Conflict of interest statement

The authors declare that the research was conducted in the absence of any commercial or financial relationships that could be construed as a potential conflict of interest.

## References

[B1] Almeida N. F. Yan S. Cai R. Clarke C. R. Morris C. E. Schaad N. W.. (2010). PAMDB, a multilocus sequence typing and analysis database and website for plant-associated microbes. Phytopathology 100, 208–215. 10.1094/PHYTO-100-3-0208 20128693

[B2] Altschul S. F. Madden T. L. Schäffer A. A. Zhang J. Zhang Z. Miller W.. (1997). Gapped BLAST and PSI-BLAST: a new generation of protein database search programs. Nucleic Acids Res. 25, 3389–3402. 10.1093/nar/25.17.3389 9254694 PMC146917

[B3] Astua-Monge G. Minsavage G. V. Stall R. E. Davis M. J. Bonas U. Jones J. B. (2000). Resistance of tomato and pepper to T3 strains of *Xanthomonas campestris* pv. *vesicatoria* is specified by a plant-inducible avirulence gene. Mol. Plant Microbe Interact. 13, 911–921. 10.1094/MPMI.2000.13.9.911 10975648

[B4] Bart R. Cohn M. Kassen A. McCallum E. J. Shybut M. Petriello A.. (2012). High-throughput genomic sequencing of cassava bacterial blight strains identifies conserved effectors to target for durable resistance. Proc. Natl. Acad. Sci. U.S.A. 109, E1972–E1979. 10.1073/pnas.1208003109 22699502 PMC3396514

[B3a] Basim H. Stall R. E. Minsavage G. V. Jones J. B. (1999). Chromosomal gene transfer by conjugation in the plant pathogen *Xanthomonas axonopodis* pv. *vesicatoria*. Phytopathology 89, 1044–1049. 10.1094/PHYTO.1999.89.11.1044 18944660

[B5] Bolger A. M. Lohse M. Usadel B. (2014). Trimmomatic: a flexible trimmer for Illumina sequence data. Bioinformatics 30, 2114–2120. 10.1093/bioinformatics/btu170 24695404 PMC4103590

[B6] Boyd L. A. Ridout C. O'Sullivan D. M. Leach J. E. Leung H. (2013). Plant-pathogen interactions: disease resistance in modern agriculture. Trends Genet. 29, 233–240. 10.1016/j.tig.2012.10.011 23153595

[B7] Contreras-Moreira B. Vinuesa P. (2013). GET_HOMOLOGUES, a versatile software package for scalable and robust microbial pangenome analysis. Appl. Environ. Microbiol. 79, 7696–7701. 10.1128/AEM.02411-13 24096415 PMC3837814

[B9] da Silva A. C. R. Ferro J. A. Reinach F. C. Farah C. S. Furlan L. R. Quaggio R. B.. (2002). Comparison of the genomes of two *Xanthomonas* pathogens with differing host specificities. Nature 417, 459–463. 10.1038/417459a 12024217

[B10] Grant S. R. Fisher E. J. Chang J. H. Mole B. M. Dangl J. L. (2006). Subterfuge and manipulation: type III effector proteins of phytopathogenic bacteria. Annu. Rev. Microbiol. 60, 425–449. 10.1146/annurev.micro.60.080805.142251 16753033

[B11] Hajri A. Brin C. Hunault G. Lardeux F. Lemaire C. Manceau C.. (2009). A “repertoire for repertoire” hypothesis: repertoires of type three effectors are candidate determinants of host specificity in *Xanthomonas*. PLoS ONE 4:e6632. 10.1371/journal.pone.0006632 19680562 PMC2722093

[B12] Hamza A. A. Robène-Soustrade I. Jouen E. Gagnevin L. Lefeuvre P. (2010). Genetic and pathological diversity among *Xanthomonas* strains responsible for bacterial spot on tomato and pepper in the southwest Indian Ocean region. Plant Dis. 94, 993–999. 10.1094/PDIS-94-8-0993 30743480

[B13] Hert A. P. Roberts P. D. Momol M. T. Minsavage G. V. Tudor-Nelson S. M. Jones J. B. (2005). Relative importance of bacteriocin-like genes in antagonism of *Xanthomonas perforans* tomato race 3 to *Xanthomonas euvesicatoria* tomato race 1 strains. Appl. Environ. Microbiol. 71, 3581–3588. 10.1128/AEM.71.7.3581-3588.2005 16000765 PMC1168993

[B14] Horvath D. M. Stall R. E. Jones J. B. Pauly M. H. Vallad G. E. Dahlbeck D.. (2012). Transgenic resistance confers effective field level control of bacterial spot disease in tomato. PLoS ONE 7:e42036. 10.1371/journal.pone.0042036 22870280 PMC3411616

[B15] Ibarra Caballero J. Zerillo M. M. Snelling J. Boucher C. Tisserat N. (2013). Genome sequence of *Xanthomonas arboricola* pv. corylina, isolated from Turkish filbert in Colorado. Genome Announc. 1, e00246–e00213. 10.1128/genomeA.00246-13 23704178 PMC3662818

[B16] Jalan N. Aritua V. Kumar D. Yu F. Jones J. B. Graham J. H.. (2011). Comparative genomic analysis of *Xanthomonas axonopodis* pv. *citrumelo* F1, which causes citrus bacterial spot disease, and related strains provides insights into virulence and host specificity. J. Bacteriol. 189, 6342–6357. 10.1128/JB.05777-11 21908674 PMC3209208

[B17] Jones J. B. Bouzar H. Somodi G. C. Stall R. E. Pernezny K. El-Morsy G.. (1998b). Evidence for the preemptive nature of tomato race 3 of *Xanthomonas campestris* pv. *vesicatoria* in Florida. Phytopathology 88, 33–38. 10.1094/PHYTO.1998.88.1.33 18944996

[B18] Jones J. B. Lacy G. H. Bouzar H. Stall R. E. Schaad N. W. (2004). Reclassification of the xanthomonads associated with bacterial spot disease of tomato and pepper. Syst. Appl. Microbiol. 27, 755–762. 10.1078/0723202042369884 15612634

[B19] Jones J. B. Stall R. E. (2000). Systematic analysis of xanthomonads (*Xanthomonas* spp.) associated with pepper and tomato lesions. Int. J. Syst. Evol. Microbiol. 50, 1211–1219. 10.1099/00207713-50-3-1211 10843065

[B20] Jones J. B. Stall R. E. Bouzar H. (1998a). Diversity among xanthomonads pathogenic on pepper and tomato. Annu. Rev. Phytopathol. 36, 41–58. 10.1146/annurev.phyto.36.1.41 15012492

[B21] Jones J. D. G. Dangl J. L. (2006). The plant immune system. Nature 444, 323–329. 10.1038/nature05286 17108957

[B22] Katoh K. Misawa K. Kuma K.-I. Miyata T. (2002). MAFFT: a novel method for rapid multiple sequence alignment based on fast Fourier transform. Nucleic Acids Res. 30, 3059–3066. 10.1093/nar/gkf436 12136088 PMC135756

[B23] Kay S. Bonas U. (2009). How *Xanthomonas* type III effectors manipulate the host plant. Curr. Opin. Microbiol. 12, 37–43. 10.1016/j.mib.2008.12.006 19168386

[B24] Kim N. H. Choi H. W. Hwang B. K. (2010). *Xanthomonas campestris* pv. *vesicatoria* effector AvrBsT induces cell death in pepper, but suppresses defense responses in tomato. Mol. Plant Microbe Interact. 23, 1069–1082. 10.1094/MPMI-23-8-1069 20615117

[B25] Kück P. Meusemann K. (2010). FASconCAT: convenient handling of data matrices. Mol. Phylogenet. Evol. 56, 1115–1118. 10.1016/j.ympev.2010.04.024 20416383

[B26] Lanfear R. Calcott B. Ho S. Y. W. Guindon S. (2012). Partitionfinder: combined selection of partitioning schemes and substitution models for phylogenetic analyses. Mol. Biol. Evol. 29, 1695–1701. 10.1093/molbev/mss020 22319168

[B27] Lanfear R. Calcott B. Kainer D. Mayer C. Stamatakis A. (2014). Selecting optimal partitioning schemes for phylogenomic datasets. BMC Evol. Biol. 14:82. 10.1186/1471-2148-14-82 24742000 PMC4012149

[B28] Langmead B. Salzberg S. L. (2012). Fast gapped-read alignment with Bowtie2. Nat. Methods 9, 357–359. 10.1038/nmeth.1923 22388286 PMC3322381

[B29] Lee B. M. Park Y. Park D. Kang H. Kim J. Song E.. (2005). The genome sequence of *Xanthomonas oryzae* pv. *oryzae* KACC10331, the bacterial blight pathogen of rice. Nucleic Acids Res. 33, 577–586. 10.1093/nar/gki206 15673718 PMC548351

[B30] Li L. Stoeckert C. J. Jr. Roos D. S. (2003). OrthoMCL: identification of ortholog groups for eukaryotic genomes. Genome Res. 13, 2178–2189. 10.1101/gr.1224503 12952885 PMC403725

[B31] Lindgren P. B. Peet R. C. Panopoulos N. J. (1986). Gene-cluster of *Pseudomonas syringae* pv. *phaseolicola* controls pathogenicity of bean plants and hypersensitivity on nonhost plants. J. Bacteriol. 168, 512–522. 3023280 10.1128/jb.168.2.512-522.1986 PMC213511

[B32] Ma X. Lewis Ivey M. L. Miller S. A. (2011). First report of *Xanthomonas gardneri* causing bacterial spot of tomato in Ohio and Michigan. Plant Dis. 95, 1584–1584. 10.1094/PDIS-05-11-0448 30732002

[B33] McKenna A. Hanna M. Banks E. Sivachenko A. Cibulskis K. Kernytsky A.. (2010). The Genome Analysis Toolkit: a MapReduce framework for analyzing next-generation DNA sequencing data. Genome Res. 20, 1297–1303. 10.1101/gr.107524.110 20644199 PMC2928508

[B35a] Mhedbi-Hajri N. Hajri A. Boureau T. Darrasse A. Durand K. Brin C.. (2013). Evolutionary history of the plant pathogenic bacterium *Xanthomonas axonopodis*. PLoS ONE 8:e58474. 10.1371/journal.pone.0058474 23505513 PMC3591321

[B34] Midha S. Patil P. B. (2014). Genomic insights into the evolutionary origin of *Xanthomonas axonopodis* pv. *citri* and its ecological relatives. Appl. Environ. Microbiol. 80, 6266–6279. 10.1128/AEM.01654-14 25085494 PMC4178650

[B35] Minsavage G. V. Dahlbeck D. Whalen M. C. Kearney B. Bonas U. Staskawicz B. J. (1990). Gene-for-gene relationships specifying disease resistance in *Xanthomonas campestris* pv. *vesicatoria*-pepper interactions. Mol. Plant Microbe Interact. 3, 41–47. 10.1094/MPMI-3-041

[B36] Monteil C. L. Cai R. Liu H. Llontop M. E. M. Leman S. Studholme D. J.. (2013). Nonagricultural reservoirs contribute to emergence and evolution of *Pseudomonas syringae* crop pathogens. New Phytol. 199, 800–811. 10.1111/nph.12316 23692644

[B37] Moreira L. M. Almeida N. F. Potnis N. Digiampietri L. A. Adi S. S. Bortolossi J. C.. (2010). Novel insights into the genomic basis of citrus canker based on the genome sequences of two strains of *Xanthomonas fuscans* subsp. *aurantifolii*. BMC Genomics 11:238. 10.1186/1471-2164-11-238 20388224 PMC2883993

[B38] Nguyen L.-T. Schmidt H. A. von Haeseler A. Minh B. Q. (2015). IQ-TREE: a fast and effective stochastic algorithm for estimating maximum-likelihood phylogenies. Mol. Biol. Evol. 32, 268–274. 10.1093/molbev/msu300 25371430 PMC4271533

[B39] Obradovic A. Jones J. B. Balogh B. Momol M. T. (2008). Integrated management of tomato bacterial spot, in Integrated Management of Disease Caused by Fungi, Phytoplasma, and Bacteria, Integrated Management of Plant Pests and Diseases, eds Ciancio A. Mukerji K. G. (Dordecht: Springer Science+Business Media), 211–223.

[B40] Parkinson N. M. Cowie C. Heeney J. Stead D. E. (2009). Phylogenetic structure of *Xanthomonas* determined by comparison of *gyrB* sequences. Int. J. Syst. Evol. Microbiol. 59, 264–274. 10.1099/ijs.0.65825-0 19196764

[B41] Penn O. Privman E. Ashkenazy H. Landan G. Graur D. Pupko T. (2010). GUIDANCE: a web server for assessing alignment confidence scores. Nucleic Acids Res. 38, 23–28. 10.1093/nar/gkq443 20497997 PMC2896199

[B42] Potnis N. Krasileva K. Chow V. Almeida N. F. Patil P. B. Ryan R. P.. (2011). Comparative genomics reveals diversity among xanthomonads infecting tomato and pepper. BMC Genomics 12:146. 10.1186/1471-2164-12-146 21396108 PMC3071791

[B43] Potnis N. Minsavage G. Smith J. K. Hurlbert J. C. Norman D. Rodrigues R.. (2012). Avirulence proteins AvrBs7 from *Xanthomonas gardneri* and AvrBs1.1 from *Xanthomonas euvesicatoria* contribute to a novel gene-for-gene interaction in pepper. Mol. Plant Microbe Interact. 25, 307–320. 10.1094/MPMI-08-11-0205 22112215

[B44] Schornack S. Minsavage G. V. Stall R. E. Jones J. B. Lahaye T. (2008). Characterization of AvrHah1, a novel AvrBs3-like effector from *Xanthomonas gardneri* with virulence and avirulence activity. New Phytol. 179, 546–556. 10.1111/j.1469-8137.2008.02487.x 19086184

[B45] Stall R. E. Beaulieu C. Egel D. Hodge N. C. Leite R. P. Minsavage G. V. (1994). Two genetically diverse groups of strains are included in *Xanthomonas campestris* pv. *vesicatoria*. Int. J. Syst. Bacteriol. 44, 47–53. 10.1099/00207713-44-1-47

[B46] Stall R. E. Jones J. B. Minsavage G. V. (2009). Durability of resistance in tomato and pepper to xanthomonads causing bacterial spot. Annu. Rev. Phytopathol. 47, 265–284. 10.1146/annurev-phyto-080508-081752 19400644

[B47] Stamatakis A. (2014). RAxML Version 8: a tool for phylogenetic analysis and post-analysis of large phylogenies. Bioinformatics 30, 1312–1313. 10.1093/bioinformatics/btu033 24451623 PMC3998144

[B48] Sugio A. Yang B. White F. F. (2005). Characterization of the *hrpF* pathogenicity peninsula of *Xanthomonas oryzae* pv. *oryzae*. Mol. Plant Microbe Interact. 18, 546–554. 10.1094/MPMI-18-0546 15986924

[B49] Swords K. Dahlbeck D. Staskawicz B. J. (1996). Spontaneous and induced mutations in a single open reading fram alter both virulence and avirulence in *Xanthomonas campestris* pv. *vesicatoria avrBs2*. J. Bacteriol. 178, 4661–4669. 8755898 10.1128/jb.178.15.4661-4669.1996 PMC178237

[B50] Tamura K. Stecher G. Peterson D. Filipski A. Kumar S. (2013). MEGA6: molecular evolutionary genetics analysis version 6.0. Mol. Biol. Evol. 30, 2725–2729. 10.1093/molbev/mst197 24132122 PMC3840312

[B51] Thieme F. Koebnik R. Bekel T. Berger C. Boch J. Büttner D.. (2005). Insights into genome plasticity and pathogenicity of the plant pathogenic bacterium *Xanthomonas campestris* pv. *vesicatoria* revealed by the complete genome sequence. J. Bacteriol. 187, 7254–7266. 10.1128/JB.187.21.7254-7266.2005 16237009 PMC1272972

[B52] Timilsina S. Jibrin M. O. Potnis N. Minsavage G. V. Kebede M. Schwartz A. (2015). Multilocus sequence analysis of xanthomonads causing bacterial spot of tomato and pepper plants reveals strains generated by recombination among species and recent global spread of *Xanthomonas gardneri*. Appl. Environ. Microbiol. 81, 1520–1529. 10.1128/AEM.03000-14 PMC4309686 25527544

[B53] Tudor-Nelson S. M. Minsavage G. V. Stall R. E. Jones J. B. (2003). Bacteriocin-Like Substances from Tomato Race 3 Strains of *Xanthomonas campestris* pv. *vesicatoria*. Phytopathology 93, 1415–1421. 10.1094/PHYTO.2003.93.11.1415 18944070

[B54] Vandroemme J. Cottyn B. Baeyen S. De Vos P. Maes M. (2013). Draft genome sequence of *Xanthomonas fragariae* reveals reductive evolution and distinct virulence-related gene content. BMC Genomics 14:829 10.1186/1471-2164-14-829 24274055 PMC4046712

[B55] Vauterin L. Hoste B. Kersters K. Swings J. (1995). Reclassification of *Xanthomonas*. Int. J. Syst. Bacteriol. 45, 472–489. 10.1099/00207713-45-3-472

[B56] Wasukira A. Tayebwa J. Thwaites R. Paszkiewicz K. Aritua V. Kubiriba J.. (2012). Genome-wide sequencing reveals two major sub-lineages in the genetically monomorphic pathogen *Xanthomonas campestris* pv. *musacearum*. Genes 3, 361–377. 10.3390/genes3030361 24704974 PMC3902798

[B57] Wei C.-F. Kvitko B. H. Shimizu R. Crabill E. Alfano J. R. Lin N.-C.. (2007). A *Pseudomonas syringae* pv. *tomato* DC3000 mutant lacking the type III effector HopQ1-1 is able to cause disease in the model plant *Nicotiana benthamiana*. Plant J. 51, 32–46. 10.1111/j.1365-313X.2007.03126.x 17559511

[B58] Wichmann G. Ritchie D. Kousik C. S. Bergelson J. (2005). Reduced genetic variation occurs among genes of the highly clonal plant pathogen *Xanthomonas axonopodis* pv. *vesicatoria*, including the effector gene *avrBs2*. Appl. Environ. Microbiol. 71, 2418–2432. 10.1128/AEM.71.5.2418-2432.2005 15870329 PMC1087534

[B59] Young J. M. Park D. C. Shearman H. M. Fargier F. (2008). A multilocus sequence analysis of the genus *Xanthomonas*. Syst. Appl. Microbiol. 31, 366–377. 10.1016/j.syapm.2008.06.004 18783906

