# Supplementary figures and images for: Phylogenomics of Xanthomonas field strains infecting pepper and tomato reveals diversity in effector repertoires and identifies determinants of host specificity
Source: Front Microbiol. 2015 Jun 3;6:535. doi: 10.3389/fmicb.2015.00535 (PMC4452888; doi:10.3389/fmicb.2015.00535)

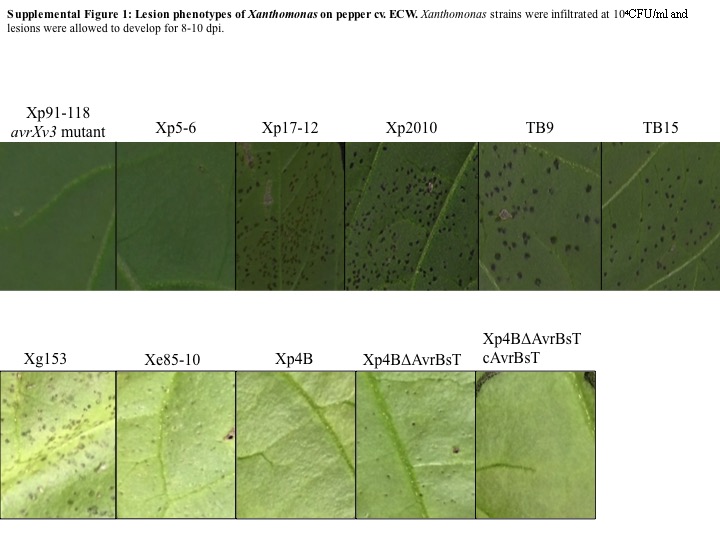

Supplement: Supplementary file 8 [file Image1.JPEG]

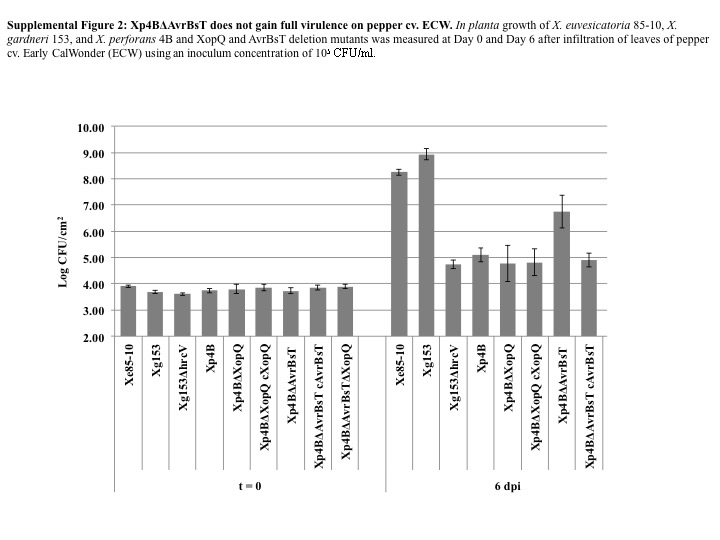

Supplement: Supplementary file 9 [file Image2.JPEG]

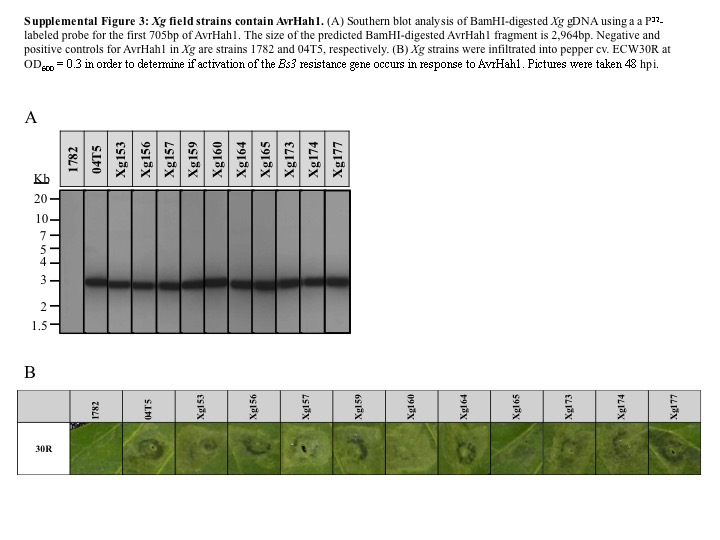

Supplement: Supplementary file 10 [file Image3.JPEG]
